# Bovine colostrum and product intervention associated with relief of childhood infectious diarrhea

**DOI:** 10.1038/s41598-019-39644-x

**Published:** 2019-02-28

**Authors:** Ji Li, Yi-Wen Xu, Jing-Jing Jiang, Qing-Kun Song

**Affiliations:** 1 Department of Pediatrics, Peking Union Medical College Hospital, Chinese Academy of Medical Sciences, Beijing, 100730 China; 2 Department of Science and Technology, Beijing Shijitan Hospital, Capital Medical University, Beijing, 100038 China; 3 Beijing Key Laboratory of Cancer Therapeutic Vaccines, Beijing, 100038 China

## Abstract

This meta-analysis aimed to investigate the protective effects of bovine colostrum against childhood infectious diarrhea. A systematic search was conducted using PubMed, Cochrane Library databases and clinicaltrial.gov. Among 166 research articles, only five RCTs were included into final analysis. Review manager (version 5.2) was used to pool the effect-size across studies. Sensitivity and risk of bias were estimated accordingly. Under a pooled analysis, bovine colostrum consumption correlated with a significant reduction in stool frequency of infectious diarrhea, by 1.42 times per day (95% CI: −2.70, −0.14). Bovine colostrum intervention also reduced occurrence of diarrhea by 71% (pooled OR = 0.29, 95%CI 0.16, 0.52). The OR of positive detection of pathogen in the stool was 0.29 (95%CI 0.08, 0.71) in bovine colostrum treated group, compared with placebo group. In the sensitivity analysis of studies with low risk of biases, bovine colostrum significantly reduced stool frequency, occurrence of diarrhea and pathogen detection. BC and related products have a significant benefit in reducing the frequency and relieving the symptoms of childhood infectious diarrhea.

## Introduction

Acute diarrhea is one of the most severe diseases with high health care costs and high mortality among children, especially in developing countries. Each year, more than 700 million children under five years are affected by acute diarrhea worldwide^1^. Approximately two to three million deaths (mainly in young children) are caused by diarrhea in developing countries annually^2^. In China, the prevalence of acute diarrhea was 5% among children younger than 5 years, with an annual incidence of 1430/100,000 per person-year^3^. About 30% of children with diarrhea were rotavirus positive^4^. Among the children with infectious diarrhea, the proportion of *Rotavirus*, *Salmonella*, *Vibrio parahaemolyticus* and *Escherichia coli* was 92%, 3%, 2% and 1%, respectively^5^.

Bovine colostrum (BC) is the first form of milk produced by a lactating dairy cow immediately following delivery of newborn calves. BC is rich in immunoglobulin, which can protect the neonatal bovine against environmental pathogens. BC is found to be effective in the prophylaxis of recurrent respiratory tract infection and diarrhea in children^6^. Infants received formula supplemented with BC products had a decreased stool frequency than those with control^7^. In contrast, another randomized control trial (RCT) presented a non-significant effect of BC supplementation on stool frequency^8^. Since diarrhea is a serious disease burden in children, while BC products show heterogeneous effects on this disease, we designed a meta-analysis using RCTs, to investigate whether BC products exert a beneficial effect against infectious diarrhea among children.

## Results

**Figure 1:**
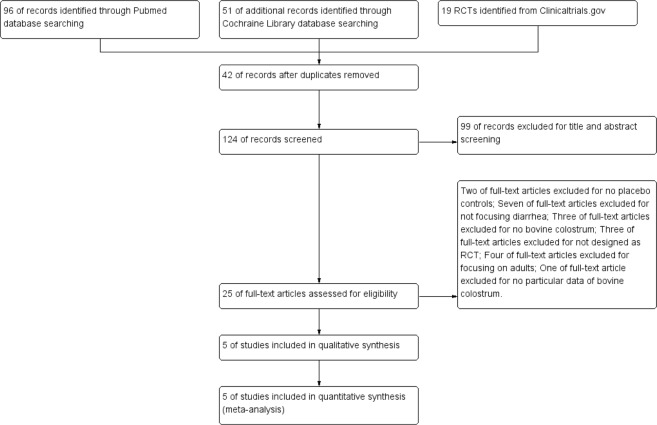
Flow chart of paper searching.

96, 51 papers and 19 trials, respectively, were searched from PubMed, Cochraine Library databases and clinicaltrials.gov (Fig. 1). 42 duplicates were removed and 99 papers were excluded after double-check on the titles and abstracts (Fig. 1). Based on detailed full-text reading, 2 articles without controls, 7 articles not focusing on diarrhea, 3 articles without using BC, 3 articles not designed as RCT, 4 articles recruiting adults, and 1 study not providing the data of BC were excluded from final analysis (Fig. 1). Finally, 5 articles in the design of RCT were included into the analysis (Fig. 1).

All of the included studies were designed as RCT. Three studies investigated the effects of BC or related product against diarrhea due to *rotavirus* and another two investigated the protective effects against *E*. *coli* (Table 1). The outcome included stool frequency, detection of pathogen in the stool and the number of patients with diarrhea at the end of the study (Table 1). Totally, 324 children were included in this meta-analysis (Table 1).

**Table 1:** The characteristics of included RCT studies.

Author	Year	Area	Participants	Intervention	Control	Outcome	Sample size
Huppertz *et al*.	1999	Germany	Children with diarrhea of *E*. *coli*	Bovine colostrum	Placebo	Stool frequency	27
Tawfeek *et al*.	2003	Iraq	Healthy infants	Immunoglobulin from hyperimmune bovine colostrum against *E*. *coli*	Placebo	Stool frequency, Detection of *E*. *coli* in the stool	84
Davidson *et al*.	1989	Australia	Children admitted into the hospital	Hyperimmune bovine colostrum against *rotavirus*	Placebo	Number of patients with diarrhea at the end of the study, Detection of *rotavirus* in the stool	120
Ebina *et al*.	1985	Japan	Children with diarrhea of *rotavirus*	Hyperimmune bovine colostrum against *rotavirus*	Placebo	Number of patients with diarrhea at the end of the study, Detection of rotavirus in the stool	13
Sarker *et al*.	1998	Sweden	Children with diarrhea of *rotavirus*	Immunoglobulin from hyperimmune bovine colostrum against *rotavirus*	Placebo	Stool frequency, Number of patients with diarrhea at the end of the study, Detection of *rotavirus* in the stool	80

RCT: randomized control trial.

**Figure 2:**
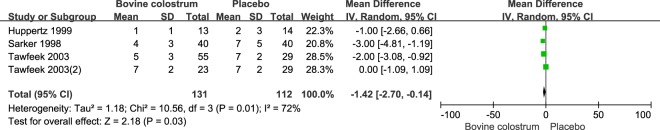
Pooled effect of bovine colostrum on frequency of stool.

**Figure 3:**
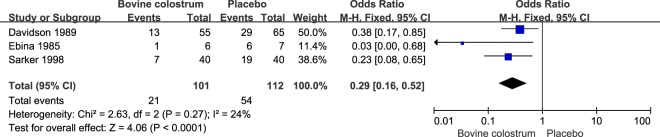
Pooled effect of bovine colostrum on diarrhea after intervention.

**Figure 4:**
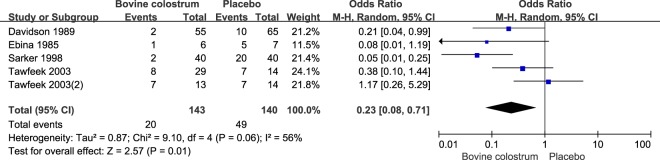
Pooled effect of bovine colostrum on positive detection of pathogen in the stool.

Four studies presented a protective effect from BC consumption against stool frequency per day. Stool frequency was reduced by 1.42 (95% CI: −2.70, −0.14) times per day under the random model (Fig. 2). Three studies focused the effects of BC against occurrence of diarrhea at the end of the study, with a pooled OR of 0.29 (95% CI: 0.16, 0.52) (Fig. 3). Five studies showed a protective effect of BC on the detection of pathogen in the stool (Fig. 4). Positive detection of pathogen in the stool was reduced by 77% (pooled OR = 0.23, 95% CI: 0.12, 0.43) (Fig. 4).

**Figure 5:**
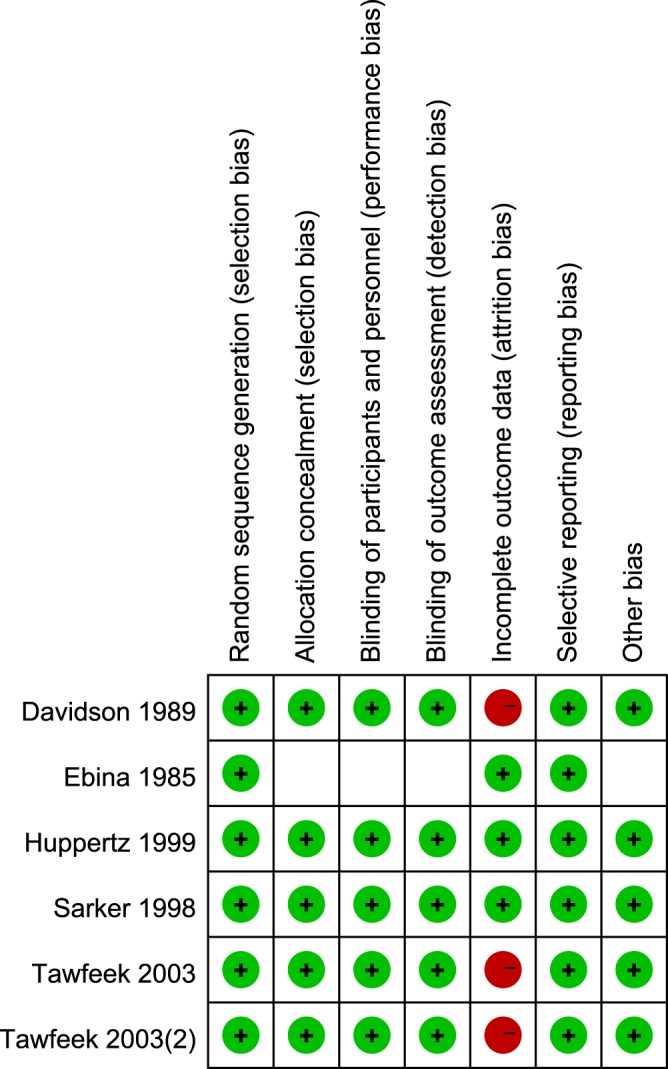
Risk biases in included studies.

**Figure 6:**
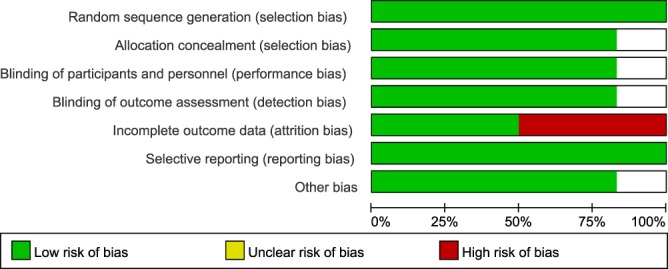
Percentage of risk biases in included studies.

Two studies had a high risk of bias due to incomplete data on clinical outcome, while two studies had a low risk of bias (Fig. 5). All of the included studies had a low risk of selection bias in random sequence generation and selective reporting (Fig. 6). A low risk of bias in allocation concealment, blinding of participants and personnel, and blinding of outcome assessment was observed in 80% of studies (Fig. 6). Attrition bias in incomplete data on outcome was showed in 50% of studies (Fig. 6). Based on the two studies with a low risk of bias in the whole research process (Huppertz 1999 and Sarker 1998), BC and related products consumption exerted a significantly pooled effect against stool frequency, diarrhea and pathogen detection in the stool (Table 2).

**Table 2:** Sensitivity analysis among studies with low risk of biases.

Outcome	Number of studies	Overall effect	p-value for heterogeneity	Model
Stool frequency	2	−1.92 (−3.14, −0.69)	0.11	Fixed model
Diarrhea	1	0.23 (0.08, 0.65)	—	Fixed model
Pathogen in the stool	1	0.05 (0.01, 0.65)	—	Fixed model

## Discussion

Under the systematic search, five RCTs were included into this meta-analysis. 324 children were analyzed to evaluate the effects of BC (products) on the outcomes of infectious diarrhea, in terms of stool frequency, occurrence of diarrhea and detection of pathogen in the stool. Compared with placebo, BC products were effective to reduce frequency of stool, occurrence of diarrhea at the end of intervention and positive detection of *rotavirus* and *E*. *coli* in stool.

Children received a four-week BC treatment and presented a significant reduction in the episodes of respiratory tract infection, diarrhea and hospitalization^6^. Even in RCTs, BC showed a protective effect on upper respiratory tract infection^9^. The children with nonorganic failure to thrive received a three-month BC treatment and had a significant body weight increase^10^. BC has the potential to relieve infection and improve the growth of children. However, a three-week supplementation of BC to neonates with very low birth weight, failed in prophylaxis on necrotizing enterocolitis and sepsis^11^. BC, hyperimmune BC as well as immunoglobulin from hyperimmune BC had substantial benefits on childhood infectious diarrhea^7,8,12–14^. Bovine immunoglobulins are promising approches to enhance children immune function, such as phagocytosis, killing of bacteria, antigen presentation and gastrointestinal barrier function^15^. In addition, the hyperimmune BC was produced by vaccinating pregnant bovines with strains of particular pathogens. Hyperimmune BC was more effective than ordinary BC against diarrhea due to *rotavirus*^16^, but not the diarrhea due to *shigellosis*^17^.

Mice administered with BC showed a reduction of intestinal damages and clinical signs of colitis induced by 2,4,6 trinitrobenzene sulfonic acid. Accordingly, TLR4, IL-1β, IL-8 and IL-10 were downregulated^18^. Colostrum supplementation enhanced NK cell cytotoxicity and promoted immune response to primary *influenza* virus infection in mice^19^. Compared with milk-supplement, colostrum supplement treated mice had an increase in IL-6 production, as well as IgA production derived from B cells in small intestine and lung^19^. Even to preterm pigs, BC formula was advantageous in the prevention of gut dysfunction, necrotizing enterocolitis, and systemic infection^20^. There might be an interaction between BC and immunity at the intestinal epithelium.

In this meta-analysis, children with diarrhea were administered with BC in one study, hyperimmune BC in two studies, or immunoglobulin from hyperimmune BC in another two studies. Hyperimmune BC had significant effects on reduced diarrhea occurrence (OR = 0.32, 95% CI: 0.15, 0.67). Immunoglobulin from hyperimmune BC also reduced stool frequency, diarrhea occurrence and positive pathogen detection. BC was effective to reduce stool frequency by once per day (95% CI: −2.66, 0.66). In the analysis of pooled effect on stool frequency, there was a significant heterogeneity between studies and the heterogeneity was introduced by the study of Tawfeek(2). When we conducted the sensitivity analysis of excluding Tawfeek(2)’ study, BC intervention was still significant to reduce the stool frequency and the heterogeneity was non-significant.

The limited sample size was the major limitation of this study. The merge of BC, hyperimmune BC and derived immunoglobulin was another limitation in this meta-analysis.

## Conclusion

BC products were effective to control clinical symptoms and pathogenic agents, in terms of stool frequency, diarrhea occurrence and positive pathogen detection, of infectious diarrhea among children. From this meta-analysis, it is meaningful to promote the application of BC products among children with infectious diarrhea.

## Methods

### Databases

We searched articles from PubMed Database (http://www.ncbi.nlm.nih.gov/pubmed/), Cochrane Library Database (http://onlinelibrary.wiley.com/cochranelibrary/search) and clinicaltrials.gov (https://www.clinicaltrials.gov/ct2/results?term=colostrum&Search=Apply&recrs=g&recrs=h&recrs=e&recrs=i&age_v=&age=0&gndr=&type=Intr&rslt=). Participants were “children”, the intervention was “BC or product” and the control was “placebo”. The outcome included stool frequency, diarrhea, and detection of pathogen in the stool. All included studies were designed as RCT.

### Search terms and strategies

The search term was “colostrum”. In PubMed database, the additional filters were “humans”, “clinical trial”, “age < 18 years” and “published to August 31, 2018”. In Cochrane Library database, the additional filters were “trials” and “childhood health”. In clinicaltrials.gov, the additional filters were “trials”, “children (birth-17)” and “recruitment status including suspended, completed, terminated and withdrawn”.

### Included studies

The studies under the design of RCT were eligible to include in the analysis. Phase I clinical trial, observational, animal or laboratory studies were excluded from the analysis. The included studies should provide the information on stool frequency, diarrhea occurrence at the end of study, and detection of pathogen in stool. This study was focused on original RCT but not reanalysis of previous data, review, abstracts or comments.

### Data extraction and quality assessment

The number of patients with diarrhea, stool frequency, pathogen detection in the stool at the end of study, publication year, sample size and research area were extracted from the included articles by two reviewers independently. The quality assessment procedure of each article was focused on random sequence generation, allocation concealment, blinding of participants and personnel, blinding of outcome assessment, incomplete outcome data, selective reporting and other possible bias^21^. Stool frequency indicated the times of bowel movements per day. Diarrhea was defined as having four or more loose or watery stool in a 24-hour period.

### Data synthesis

Reviewer Manager 5.2 (Version 5.2.9, Copenhagen: The Nordic Cochrane Centre, The Cochrane Collaboration, 2012.) was used to estimate the overall effect and heterogeneity across included studies. Heterogeneity was estimated by Chi^2^-value and a p-value of <0.05 was set as significant level. For significant heterogeneity, overall effect was produced under random models; whereas for non-significant heterogeneity, under fixed models. Sensitivity was evaluated among studies with a low risk of bias.

## Data Availability

All data generated or analyzed during this study are included in this published article.
